# The Complex Epigenetic Panorama in the Multipartite Genome of the Nitrogen-Fixing Bacterium *Sinorhizobium meliloti*

**DOI:** 10.1093/gbe/evae245

**Published:** 2025-01-09

**Authors:** Iacopo Passeri, Lisa Cangioli, Marco Fondi, Alessio Mengoni, Camilla Fagorzi

**Affiliations:** Department of Biology, University of Florence, 50019 Sesto Fiorentino, Italy; Department of Biology, University of Florence, 50019 Sesto Fiorentino, Italy; Department of Biology, University of Florence, 50019 Sesto Fiorentino, Italy; Department of Biology, University of Florence, 50019 Sesto Fiorentino, Italy; Department of Biology, University of Florence, 50019 Sesto Fiorentino, Italy

**Keywords:** nitrogen fixation, multipartite genomes, bacterial epigenetics, bacterial genome evolution

## Abstract

In prokaryotes, DNA methylation plays roles in DNA repair, gene expression, cell cycle progression, and immune recognition of foreign DNA. Genome-wide methylation patterns can vary between strains, influencing phenotype, and gene transfer. However, broader evolutionary studies on bacterial epigenomic variation remain limited. In this study, we conducted an epigenomic analysis using single-molecule real-time sequencing on 21 strains of Sinorhizobium meliloti, a facultative plant nitrogen-fixing alphaproteobacterium. This species is notable for its multipartite genome structure, consisting of a chromosome, chromid, and megaplasmid, leading to significant genomic and phenotypic diversity. We identified 16 palindromic and nonpalindromic methylated DNA motifs, including *N*^4^-methylcytosine and *N*^6^-methyladenine modifications, and analyzed their associated methyltransferases. Some motifs were methylated across all strains, forming a core set of epigenomic signatures, while others exhibited variable methylation frequencies, indicating a dispensable (shell) epigenome. Additionally, we observed differences in methylation frequency between replicons and within coding sequences versus regulatory regions, suggesting that methylation patterns may reflect multipartite genome evolution and influence gene regulation. Overall, our findings reveal extensive epigenomic diversity in S. meliloti, with complex epigenomic signatures varying across replicons and genomic regions. These results enhance our understanding of multipartite genome evolution and highlight the potential role of epigenomic diversity in phenotypic variation.

**Significance**

- Understanding the complexities of epigenomic modification patterns in bacteria is crucial for unraveling the mechanisms driving phenotypic diversity, but little is known about these mechanisms in prokaryotes.
- Using genome-sequencing-based methylation analysis, we examined the methylation patterns in strains of the model plant symbiotic bacterium *Sinorhizobium meliloti*.
- A broad pan-epigenome, consisting of shared and unique methylated DNA motifs, was identified, offering a novel perspective on genome diversity and bacterial strain evolution. Additionally, the uneven methylation patterns between coding and noncoding regions suggest a significant impact of DNA methylation on phenotypic diversity in bacteria.

## Introduction

Epigenetic information (viz. DNA methylation and posttranslational modification of histones) has been shown to be pivotal in the control of several biological phenomena in eukaryotes, such as cellular differentiation, development, and pathogenesis (Jones 2012). In prokaryotes, DNA methylation is the primary means of epigenetic gene regulation and has found to be involved in many molecular mechanisms, such as DNA repair, DNA–protein interactions, gene expression, cell cycle progression, and self-DNA recognition mediated by restriction–modification (R–M) systems (Sánchez-Romero and Casadesús 2020). Novel information gained in recent years on DNA methylation in prokaryotes, especially in bacteria, is delving into transcriptional regulation and the formation of phenotypic cell variants (Vasu and Nagaraja 2013; Oliveira and Fang 2021). The overall genomic methylation (methylome) is due to the activity of DNA methyltransferases (MTases), family of enzymes catalyzing the transfer of a methyl group from S-adenosyl methionine to the DNA (Sánchez-Romero and Casadesús 2020). Three different forms of DNA methylation are found in bacterial genomes due to: *N*^6^-methyladenine (6mA), which is the most prevalent form in prokaryotes; *N*^4^-methylcytosine (4mC); and C^5^-methylcytosine (5mC) (Sánchez-Romero and Casadesús 2020). Indeed, 6mA is known to be involved in many events, spanning from chromosome replication to mismatch repair, conjugal transfer, antibiotic resistance (Sánchez-Romero and Casadesús 2020) bacterial differentiation (diCenzo et al. 2022), and phenotypic plasticity as phase variation (Atack et al. 2018). Besides, stress response and drug transport have been related to the presence of 5mC (Kahramanoglou et al. 2012), as 4mC to pathogenesis (Kumar et al. 2018). Evidence is now supporting the idea that DNA methylation in bacteria may influence phenotypes as virulence and host colonization (Blow et al. 2016).

Population genomics has shown large differences for R–M systems in clinical isolates of *Staphylococcus epidermidis* and in strains of the plant growth-promoting bacterium *Bacillus velezensis* (Reva et al. 2019) where some methylation profiles were found abundant upstream from the coding sequences (CDS), suggesting regulatory effects on transcription. A recent evolutionary study conducted on *Helicobacter pylori* showed that phase-variable DNA MTase genes contribute to the variation of the methylome among isolates (Estibariz et al. 2020), indicating that bacterial phenotypic variation and adaptation to variable environmental conditions may rely on epigenetic modification too. Other studies have shown that the genomic levels of 5mC relate to antibiotic resistance (Militello et al. 2014). More recently, a pan-epigenome analysis of *Mycoplasma agalactiae* revealed the existence of strain-specific motifs, which allowed the identification of orphan DNA MTases (Dordet-Frisoni et al. 2022), affecting the rate of horizontal gene transfer (HGT) events among *M. agalactiae* strains. Consequently, it is becoming highly relevant for molecular evolutionary studies in bacteria to investigate the extent of the pan-epigenome and evaluate its role in genome evolution and adaptation.

Facultative symbiotic bacteria are relevant models for the study of environmental adaptation, since they colonize multiple environments coping with dramatically contrasting selective pressure. *Sinorhizobium meliloti* is a facultative plant symbiotic bacterium, belonging to the *Rhizobiaceae* family, able to thrive on soil, colonizing the surrounding of plant roots and entering in symbiosis with host plants (mainly from the Leguminous genera *Medicago*, *Melilotus*, and *Trigonella*), where it establishes an intracellular symbiosis in specialized root organs called nodules, differentiates into bacteroids and fixes atmospheric dinitrogen into ammonia (Poole et al. 2018, Fagorzi et al. 2021). *Sinorhizobium meliloti* is one of the most studied species in symbiotic nitrogen fixation (diCenzo et al. 2019a, 2019b). The genomes of several *S. meliloti* strains have been sequenced and many population-genomic and evolutionary studies have been performed on this species in relation to adaptation, selective pressure for symbiosis (Batstone et al. 2020, 2022; Epstein et al. 2023) and multipartite genome evolution (Galardini et al. 2013; Schulte et al. 2022). In fact, as for many bacterial species interacting with hosts, the strains are generally composed by three main replicons (diCenzo and Finan 2017; diCenzo et al. 2019a, 2019b): a chromosome, a chromid, and a megaplasmid. The latter usually contains the machinery essential for symbiosis, whereas the other two carry essential genes for growth and survival in soil and in the plant rhizosphere (diCenzo et al. 2014; Schulte et al. 2022). Moreover, many strains have additional secondary replicons (smaller plasmids) that may harbor functions related to local adaptation (diCenzo and Finan 2017). As in the other alphaproteobacterial species (Zweiger et al. 1994), *S. meliloti* cell cycle is controlled by the action of the master regulator CtrA, which modulates its activity according to the methylation status on the pentanucleotide DNA motif GANTC, methylated by the orphan DNA MTase CcrM (Cell cycle regulated Methyltransferase) (Biondi et al. 2006; Fioravanti et al. 2013).

Recently, we investigated the DNA methylation profile in two strains of *S. meliloti* along the intracellular differentiation phases in the plant root nodule (diCenzo et al. 2022). In this study, we reported evidence that the methylation activity of CcrM on GANTC sites is dysregulated during symbiosis, further suggesting this dysregulation is a driving factor for intracellular differentiation of *S. meliloti* free-living cells into bacteroids. In the present work, we have identified the presence of additional methylated motifs other than GANTC sites. Interestingly, despite having a lower frequency than GANTC, many of them were present in only few of the investigated *S. meliloti* strains, suggesting the presence of a pan-epigenome composed by a shared set of methylated motifs (core epigenome) plus a few unshared or strain-specific motifs (shell epigenome).

In this work, we have also analyzed the 24 putative DNA MTases identified in the set of 21 *S. meliloti* genomes used in the study and we have found that CcrM was the only MTase in common to all the strains, while many others were strain-specific. These DNA MTases present in the shell genome fraction can be the genetic determinants of the unshared methylated motifs (viz. shell epigenome). Such motifs could be possibly related to R–M systems and determine the formation of barriers to gene flow. Indeed, in a previous work, we showed that the *hsd*R gene of *S. meliloti* 1021, coding for a putative type-I restriction enzyme, plays a role in the frequency of gene transfer from different donor strains (Gao et al. 2023), suggesting that the observed differences in DNA methylation profiles and DNA MTases in *S. meliloti* may represent barriers to gene flow. However, since there is mounting evidence for the role of DNA methylation in phenotypic variation in bacteria, we cannot exclude that both the core and the shell epigenome could impact on regulation of gene expression. *Sinorhizobium meliloti* strains, beside displaying relevant differences in genome content because of the typical open pangenome structure (Casadesùs and Low 2006), also display large phenotypic and transcriptomic variation, both in free-living growth and in symbiotic-related conditions (Loenen et al. 2014; Bellabarba et al. 2021). Thus, we now ask whether this large variability is also mirrored by genome-wide DNA methylation variation.

To address this point, we examined genome-wide methylation pattern variations in *S. meliloti* strains and (i) assessed the presence of a pan-epigenome, (ii) evaluated its relationship with the pangenome and the multireplicon genome structure and a possible effect on gene transfer (i.e. HGT), and (iii) inspected the frequency of an uneven distribution of methylated motifs with respect to coding sequences, thus indicating the possibility to relate epigenomic differences to gene regulation. Results obtained showed the presence of a wide pan-epigenome, including both 4mC and 6mA palindromic and nonpalindromic motifs. These motifs have a differential frequency in *S. meliloti* replicons and between coding and regulatory sequences, partially mirroring gene exchange rates. Taken together, our results suggest that the epigenome may impact *S. meliloti* microevolution and adaptation.

## Materials and Methods

### *Sinorhizobium meliloti* Strains, Growth Conditions, and DNA Extraction

*Sinorhizobium meliloti* strains used in this work are reported in Table 1 and listed in supplementary dataset S1, Supplementary Material online. All strains were routinely grown on TY medium. Overnight cultures of all strains were grown in 10 ml TY medium at 30 °C with shaking (130 rpm). To obtain late exponential/stationary phase samples, cultures were harvested after 24 h of growth at OD_600nm_ values of ∼1.4. In all cases, cultures were streaked on TY plates to check for contamination, and the full 10 ml culture was centrifuged (8,200 g, 10 min, 4 °C) and transferred to a 2 ml tube by resuspending in 750 µl of PowerBead Solution (Qiagen PowerLyser PowerSoil Pro KIT). DNA was isolated using Qiagen PowerLyser PowerSoil Pro KIT according to manufacturer instructions.

**Table 1:** List of *S. meliloti* strains used in the study

Strain code	Host plant (species, cultivar)	Isolation country	Genome length (bp)	Number of contigs	Reference
1A42	*M. sativa* “cv. Hamadani”	Iran	6.973.268	4	Talebi et al. (2008)
4B41	*M. sativa* “cv. Nikshahri”	Iran	6.755.292	3	Talebi et al. (2008)
7B22	*M. sativa* “cv. Nikshahri”	Iran	6.791.724	4	Talebi et al. (2008)
8A52	*M. sativa* “cv. Hamadani”	Iran	6.735.207	3	Talebi et al. (2008)
AK58	*M. falcata*	Kazakhstan	7.114.023	6	Roumiantseva et al. (2014)
AK75	*M. lupulina*	Kazakhstan	6.903.928	5	Roumiantseva et al. (2014)
AK83	*M. falcata*	Kazakhstan	7.171.952	7	Roumiantseva et al. (2014)
AE608H	*M. sativa* “cv. Estival”	Italy	7.318.861	4	Carelli et al. (2000)
AL703GG	*M. sativa* “cv. Lodi”	Italy	7.487.501	9	Carelli et al. (2000)
AL703H	*M. sativa* “cv. Lodi”	Italy	7.020.338	5	Carelli et al. (2000)
AO641M	*M. sativa* “cv. Oneida”	Italy	7.503.659	7	Carelli et al. (2000)
AO643DD	*M. sativa* “cv. Oneida”	Italy	7.442.138	4	Carelli et al. (2000)
BL225C	*M. sativa* “cv. Lodi”	Italy	6.996.973	3	Carelli et al. (2000)
CE480L	*M. sativa* “cv. Estival”	Italy	7.340.765	5	Carelli et al. (2000)
CL374FF	*M. sativa* “cv. Lodi”	Italy	7.436.312	6	Carelli et al. (2000)
CO431A	*M. sativa* “cv. Oneida”	Italy	7.001.362	3	Carelli et al. (2000)
CO438LL	*M. sativa* “cv. Oneida”	Italy	6.709.320	6	Carelli et al. (2000)
H1	*M. sativa*	Italy	6.912.818	6	Galardini et al. (2013)
NGR185	*M. sativa*	Canada	7.329.821	7	Prevost et al. (1999)
SM11	*M. sativa*	Germany	7.502.918	5	Stiens et al. (2006)
T073	*M. truncatula*	Tunisia	6.958.496	4	Porter and Simms (2014)

The original isolation host (*Medicago* spp.) and country of origin, genome length, and number of contigs obtained are reported. All strains were isolated from root nodules apart from H1, that comes from leaves.

### SMRT Genome Sequencing of *S. meliloti* Strains

SMRT sequencing was performed in house at the University of Florence using the Pacific Biosciences Sequel instrument (Eid et al. 2009). Genomic DNA was sheared to 20 Kbp using g-TUBEs (Covaris Inc., Woburn, MA, USA). Sheared DNA was treated with exonuclease to remove single-stranded ends and with DNA damage repair mix followed by the end-repair and the ligation of barcoded blunt adapters using SMRTbell Template Prep Kit 2.0 (PacBio, Menlo Park, CA, USA). Libraries were purified with AMPure PB beads (Beckman Coulter, Brea, CA, USA), and eight libraries with different barcodes were pooled at equimolar ratios and purified with AMPure PB beads. SMRTbell template libraries were prepared using a Sequel Binding Kit 3.0 (PacBio, Menlo Park, CA, USA), and sequenced on a Sequel instrument using a v3 or v4 sequencing primer, 1M v3 SMRT cells, and Version 3.0 sequencing chemistry.

Genome assembly was performed using the Pacific Biosciences SMRT Link software (Pacific Biosciences, Menlo Park, CA, USA). Briefly, raw reads were filtered using SFilter to remove short reads and reads derived from sequencing adapters. Filtered reads were assembled into contigs through the Microbial Assembly SMRT Link tool. Each contig has been aligned with the genome of *S. meliloti* 1021 (GenBank assembly accession GCA_000006965.1) using Mauve software (Darling et al. 2004) and categorized into Chromosome, pSymA-like replicon, pSymB-like replicon and “other” (when no mapping to *S. meliloti* 1021 was found) (supplementary dataset S2, Supplementary Material online). Genomes were reannotated with Prokka (V 1.12-beta). Genome sequences are deposited under NCBI BioProject Accession PRJNA681719.

### Identification of Methylated Bases and Mapping to Genomic Features

The assembled draft genomes were used as reference to identify methylated nucleotides through kinetic anal ysis of the aligned DNA sequence data with the SMRT Link software ver.8.0.0.80529 (Pacific Biosciences, Menlo Park, CA, USA; Flusberg et al. 2010), using default options; the number of mapped bases per sample is provided in supplementary dataset S2, Supplementary Material online. Downstream analyses were performed using MeStudio (Riccardi et al. 2022). MeStudio input files are the genome sequences in FASTA format, the genome annotation in GFF3 format, and the PacBio-derived output file with methylation annotations in GFF3 format. MeStudio allows to determine the motifs distribution along: (i) protein-coding gene with accordant (sense) strand (CDS), (ii) regions that fall between annotated genes (true intergenic, tIG), and (iii) regions upstream to the reading frame of a gene, with accordant strand (US) (supplementary fig. S1, Supplementary Material online) based on naive-match algorithm and matching the position of the methylated base annotated in the above mentioned Pacific Bioscience SMRT Link Analysis software-derived GFF3 file with the position of the corresponding base framed in the motif. The motifs are previously identified and reported by the Pacific Bioscience SMRT Link Analysis software. Each motif is then manually collected and annotated in a new line-delimited text file which is one of the inputs of the MeStudio pipeline. The differences in the frequencies of methylated motifs among categories were evaluated by post hoc Dunn's test and Pearson correlation.

### Phylogenetic Reconstruction and Clustering of Sequences

To construct an unrooted core gene phylogeny, the pangenome of the 21 *S. meliloti* strains was calculated using Roary (version 3.11.2) (Page et al. 2015) with a percent identity threshold of 95% (Supplementary dataset S3, Supplementary Material online). The nucleotide sequences of the 4,633 core genes (identified as those found in at least 99% of the genomes; supplementary dataset S3, Supplementary Material online) were aligned with PRANK (Löytynoja 2014) and the alignments were trimmed and concatenated. The concatenated alignment was used to construct a maximum likelihood phylogeny (the bootstrap best tree following 100 bootstrap replicates) using RAxML 8.4.2 (Stamatakis 2006) with the GTRCAT model. Phylogeny was visualized with the online iTOL webserver (Letunic and Bork 2021). The shell gene set of the 21 strains was used to create a presence/absence matrix. Hierarchical clustering was performed in R (R Core Team 2014) with *hclust* function based on Euclidean distance, with “complete” agglomeration method. DNA MTases were clustered by CD-HIT with default options at 0.7 similarity (Bailly et al. 2006).

### Statistical Methods

The methylation frequencies are calculated by normalizing (per each motif) the number of times that the motif has been found methylated over the total number of times it was found in each genome.

To compare genome phylogeny with epigenome-based relatedness, the cophenetic coefficients were calculated using the R function “cophenetic” (package: “stats”). The correlation between the coefficients has been obtained by using the “cor” (package: “stats”) function in R.

**Fig. 1.**
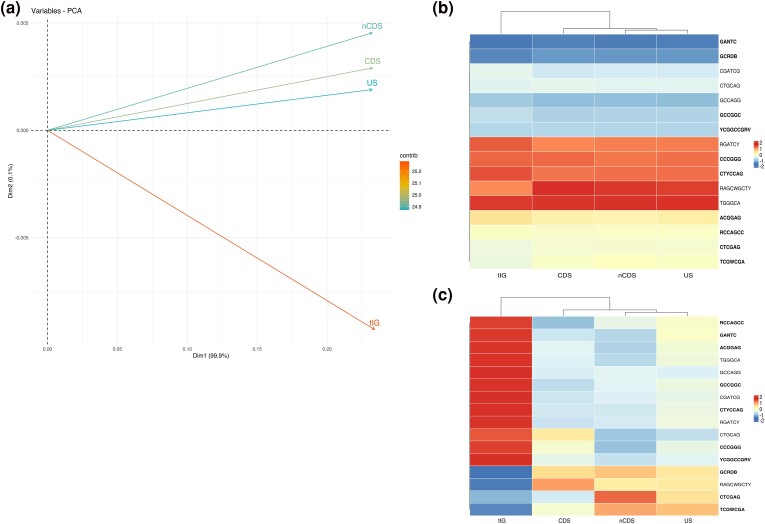
Genomic DNA regions show different methylation status. a) This plot shows the variance explained by principal components (PCs) in a PCA. “fviz_pca_var” (library factoextra, R) was used to visualize the variance associated with each PC rather than the actual data projection. The colors indicate the contribution of each variable to the PCs. b) NRMSD values motif-wise scaled. Notably, this approach reaffirms GANTC as the most consistently observed motif, displaying nearly zero variation across the diverse strains examined. c) NRMSD values for DNA region-wise scaled. tIG showed the highest variation, with both the highest and lowest NRMSD values. For both b) and c), the order of motifs derived from a hierarchical clustering analysis performed with the “pheatmap” R package.

A principal component analysis (PCA) was used to evaluate the contribution of genomic regions to the variance of methylation frequency. The principal components of the PCA were obtained by using the “prcomp” (package: “stats”) function in R. To assess the contribution to the variance for each genomic region, the function “fviz_pca_var” (package: “factoextra”) was used and plotted in the graph of variables (Fig. 1a).

We evaluated the strain-to-strain variation across DNA regions calculating the NRMSD (Fig. 1b and c). The NRMSD values depicted in the heatmaps are subject to scaling row-wise and column-wise. This approach is employed to accentuate distinct patterns of motifs across genomic features, considering the high order of magnitude in-between data. Row-wise scaling allows to appreciate the variation (or absence of variation) between the motifs, while column-wise scaling allows to appreciate the variation (or absence of variation) between the features.

Clustering of methylated motifs frequencies was obtained using the “hclust” function (package: “stats”, method: “complete”) in R. The image was then obtained applying the “as.dendrogram” function to the hclust object and using the “plot” function in R.

Statistically significant pairwise differences were calculated using the Dunn test performed in R with the function “dunn_test” (package: “rstatix”).

## Results and Discussion

### *Sinorhizobium meliloti* Harbors a Wide Pan-Epigenome Which May Affect Gene Transfer

**Fig. 2.**
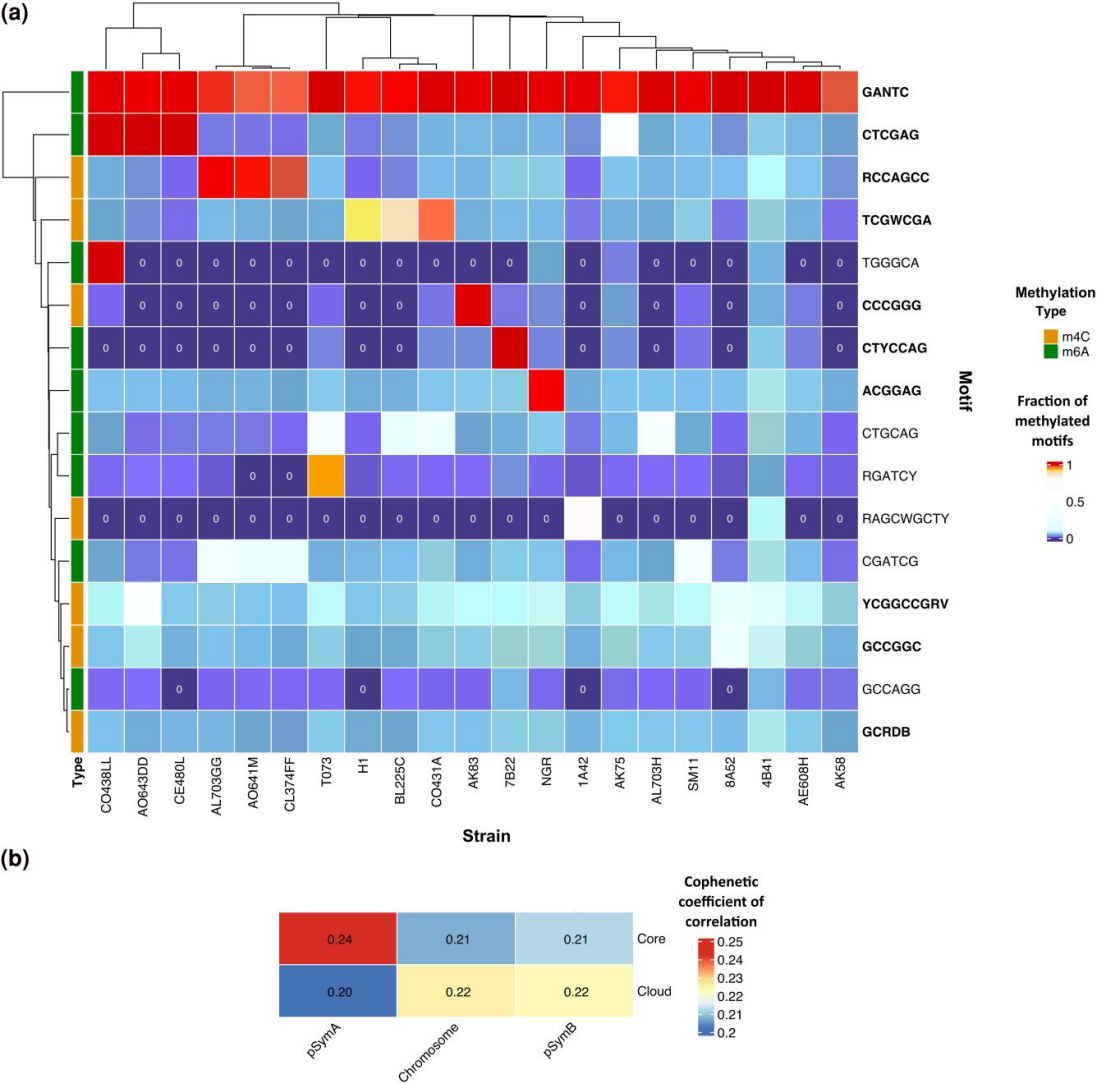
*S. meliloti* pan-epigenome partially reflects strains genomic relatedness. a) Heatmap reports the fraction of methylated motifs in each strain. Core motifs are written in bold. Cells with a value of zero (absence of methylation) are indicated. Both rows and columns are clustered according to the hclust function of “pheatmap” R package. b) Heatmap representing the cophenetic coefficients of correlation between core and shell epigenome compared to the replicons (chromosome, pSymA, and pSymB).

We sequenced 21 *S. meliloti* strains via Pacific Biosciences single-molecule real-time (SMRT) technology, collected over a wide geographical range and for which previous studies indicated a gradient of genomic diversity (Galardini et al. 2013). Genome sequences had an average coverage of 155X, an average genome size of 7.1 Mbp, and harbored from 3 to 9 contigs (for more information about the strains origin and genome organization, please refer to supplementary datasets S1 and S2, Supplementary Material online and Table 1). Mapping the sequenced data to *S. meliloti* 1021 reference genome allowed to identify a certain number of contigs not mapping to the sequences of *S. meliloti* 1021 chromosome (3.65 Mbp), pSymB chromid (1.68 Mbp), and pSymA megaplasmid (1.35 Mbp). These contigs range from few Kbp (e.g. 3,455 bp in AK75) to hundreds of Kbp (e.g. 328,353 bp in CE480L). We considered these contigs as bona-fide plasmids, additional to the three main replicons of *S. meliloti* (chromosome, pSymB, and pSymA). The presence of plasmids in *S. meliloti* was already shown for AK83 and SM11 strains (Casadesùs et al. 2006; Li et al. 2012) and it is in line with the number of genes coding for the replication initiation protein RepC identified in the genome sequences of the 21 *S. meliloti* strains (supplementary table S1, Supplementary Material online). Considering the entire panel of strains, a total of 27 methylated DNA motifs were predicted by SMRT Link software. These were further collapsed into 16 different DNA motifs (Table 2, supplementary table S2, Supplementary Material online) since they shared a common core sequence. Both 4mC (7) and 6mA (9) motifs were found as methylation types. Ten motifs were palindromic or nearly palindromic. The proportion of motifs methylated over the total number of motifs identified ranged from 0.02 to nearly fully methylated (0.99 for GANTC) (supplementary dataset S4, Supplementary Material online). The nearly full methylation for GANTC is in line with the expectation from cells collected in the late exponential growth phase, as previously observed (diCenzo et al. 2022). Notably, seven motifs presented no match to existing recognition sequences in the REBASE repository (http://rebase.neb.com/rebase/rebase.html). This observation may indicate the presence of a diverse range of DNA methylation systems within environmental prokaryotes, which have yet to be investigated. Next, we performed a methylation analysis over these 16 motifs using the MeStudio software, as explained in the “Materials and Methods” section. Notably, no strain-specific motifs were found. Out of the 16 motifs, ten exhibited methylation across all strains, whereas six motifs displayed methylation exclusively in a few strains, as reported in Fig. 2. We designated the set of ten methylated motifs common to all the strains as “core motifs” (core epigenome), and the remaining six strain-specific methylated motifs as “shell motifs” (shell epigenome), in alignment with the classifications commonly established in genomic studies (Collins and Higgs 2012). The six methylated motifs composing the shell epigenome occurred in a few as two to a maximum of 19 strains. No strain-exclusive methylated motif was found (Fig. 2, supplementary dataset S4, Supplementary Material online).

**Table 2:** Summary of DNA methylation motifs present in *S. meliloti*

Motif	Modification type	Methylated motif distribution	Motif in REBASE	Average methylation frequency
ACGGAG	m6A	Core	No	0.0872
CCCGGG	m4C	Core	Yes	0.0595
CGATCG	m6A	Shell	Yes	0.0864
CTCGAG	m6A	Core	Yes	0.1927
CTGCAG	m6A	Shell	Yes	0.0849
CTYCCAG	m6A	Core	No	0.0569
GANTC	m6A	Core	Yes	0.9913
GCCAGG	m6A	Shell	Yes	0.0162
GCCGGC	m4C	Core	Yes	0.0605
GCRDB	m4C	Core	No	0.0417
RAGCWGCTY	m4C	Shell	No	0.0384
RCCAGCC	m4C	Core	No	0.1831
RGATCY	m6A	Shell	Yes	0.0564
TCGWCGA	m4C	Core	Yes	0.1625
TGGGCA	m6A	Shell	No	0.0536
YCGGCCGRV	m4C	Core	No	0.0948

Here the 16 motifs identified by SMRT sequencing are listed. The type of modification (m6A or m4C), as well as the distribution of the motifs within the genomes (core, if methylated in all the strains, or shell, if methylated in only some of the strains), is indicated. The presence of the motifs in the REBASE database is reported. The “Average Methylation Frequency” column represents the ratio between the number of methylations found for that motif over the total times it was found along the genomes.

The core motif GANTC is the one recognized by the highly conserved cell cycle-regulated CcrM MTases. This motif was expected to be part of the core epigenome, since *ccrM* is part of the core genome, as previously reported (diCenzo et al. 2022). Analogously, we hypothesize that the other core motifs are linked to a common set of DNA MTases shared between the strains, while the shell epigenome suggests the presence of DNA MTases encoded by genes belonging to the shell genome. Genome annotation identified from 4 to 9 DNA MTases (supplementary table S3 and dataset S3, Supplementary Material online), a finding that is in line with the variability in methylated DNA motifs found among strains.

Given the observed variability, we conducted a cluster analysis using the CD-HIT (Fu et al. 2012) tool on the annotated DNA MTases, resulting in the identification of 32 distinct groups (threshold for identity: 0.7). Some DNA MTases groups were ubiquitously present across all strains (e.g. group 18, corresponding to CcrM), while others were uniquely identified in specific strains, as reported in supplementary dataset S5, Supplementary Material online. This observation prompts the hypothesis that diverse DNA MTase groups may serve distinct functional roles, potentially targeting different DNA motifs. Indeed, the clustering of strains based on DNA MTases groups (supplementary fig. S2, Supplementary Material online) presents some similarity with clustering based on methylation profile (e.g. AL703GG, AO641M, CL374FF, BL225C, and CO431A). This consistency suggests that orthologous DNA MTases may exhibit a propensity to target similar motifs. Nevertheless, the presence of strain-exclusive DNA MTase groups, coupled with the absence of strain-exclusive methylated motifs, allows to further hypothesize that different DNA MTases may target the same motif. Alternatively, and perhaps more plausibly, they may methylate additional bases from those identified by SMRT sequencing in this study or DNA motifs that were not active under the growth conditions applied.

**Fig. 3.**
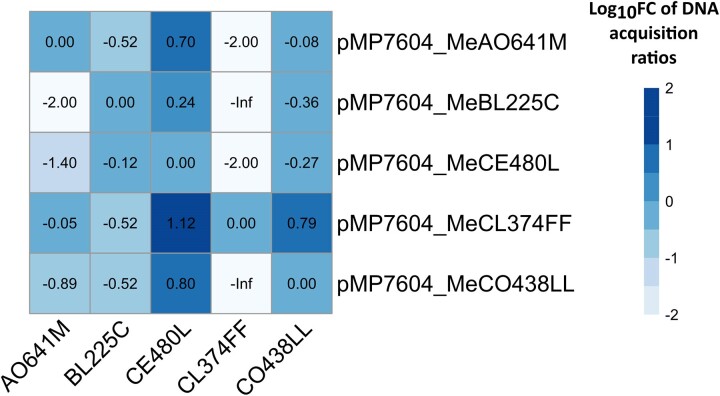
Rates of transfer of genetic material among strains. Matrix of log_10_ fold change of plasmid DNA acquisition ratios against self-DNA (rows donors, columns recipient strains). Donors are indicated with the prefix “pMP7604_Me”, where pMP7604 refers to the synthetic plasmid used and Me indicated that the plasmid DNA was methylated by the specified strain. “-Inf” indicates the absence of transformants.

Since DNA methylation may be linked to R–M systems, hence affecting gene transfer between strains, we performed an experiment to assess whether variations in DNA methylation profiles between strains (i.e. presence or absence of methylated motifs) lead to differences in the rate of genetic material transfer. We introduced a synthetic plasmid (pMP7604) into five selected strains by transformation with electroporation, a setup already reported to be informative on the effect of the R–M system on gene transfer (Gao et al. 2023). We then cultured the cells that were successfully transformed to allow them establishing their specific methylation signatures. Afterwards, we extracted the plasmid again and used it for electroporation into both the same strain and the other strains. This approach allowed us to evaluate the pairwise transfer efficiency of DNA with strain-specific methylation profiles between different strains. Results showed significant differences (up to 2 orders of magnitude) in pairwise gene transfer efficiency in five selected strains from our dataset (AO641M, BL225C, CE480L, CL374FF, and CO438LL) (Fig. 3, supplementary tables S4 to S6, Supplementary Material online), suggesting that differential presence of DNA methylated profiles could have some impact on gene transfer among strains of *S. meliloti*. For instance, the strain CL374FF was unable to be transformed with the test plasmid pMP7604 when coming from BL225C, while transformants were obtained when the plasmid was coming from other donor strains, tough showing a strong reduction in transfer rate. Similar patterns of strain-to-strain variability were observed for the other combinations, depending on both donor and recipient strains. On the contrary, the strain CL480L presented even higher transformation rates with nonself plasmid. However, it is hard to hypothesize which of the differences in DNA methylated motifs could explain such pairwise transformation efficiency.

### *Sinorhizobium meliloti* Epigenome Only Partially Mirror Strains Genomic Relatedness

Considering the presence of a core and a shell epigenome, and the effects that differences in DNA methylated profiles may have on gene transfer rates, we checked if the strains genomic relatedness of the strains was mirrored by the DNA methylation profiles. Since genomic relatedness varies between core and dispensable (shell) genome and among replicons in *S. meliloti* (Galardini et al. 2013), the analyses were performed separately between core and shell genome. For this reason, we separately clustered the DNA methylated profiles (epigenomic similarities) obtained separately from the three main replicons of *S. meliloti* (chromosome, pSymB chromid, and pSymA megaplasmid). In general, we observed that epigenome similarities are mirroring genomic relatedness and overlap with geographical distribution (e.g. for strains collected in Lodi, Italy; Galardini et al. 2013). However, some exceptions were found (AL703H and AE608H). To assess distinctions between the core and shell epigenomes across the replicons, we calculated the cophenetic correlation coefficients between the dendrograms. These coefficients measure how faithfully the compared dendrograms preserve the pairwise distances among strains. As illustrated in Fig. 2b, both the core and shell epigenomes exhibit partial correlation with strain phylogeny: in general, the shell epigenome showed higher correlation coefficients than the core epigenome. Notably, pSymA exhibited the highest cophenetic values when compared to the other replicons in both core and shell comparisons. This phylogenetic signal might imply that pSymA phylogeny is more strongly related to DNA methylation patterns, suggesting that pSymA mobility among strains, as well as acquisition of novel genomic regions on pSymA, could be highly impacted by DNA MTases linked to R–M systems harbored on pSymA itself. The phylogenetic uncoupling of pSymA from the rest of the genome in *S. meliloti* was already observed (Bailly et al. 2006). These results support the previously reported hypothesis that the distinct population structures found can result from differences in effective gene flow (Riley et al. 2023) and these are due to different pSymA geno- and methylome-types.

On the other side, the epigenome similarities shared by the three main replicons (chromosome, pSymA, and pSymB) but unshared with the additional replicons (plasmids), may likely reflect the recent origin of such additional replicons and the high rate of HGT which may lead to the acquisition of new DNA methylation profiles by novel DNA MTases harbored by such replicons.

### Epigenomic Signatures Are Unevenly Distributed with Respect to Coding Sequences

Since we hypothesized a role of DNA methylation in differential gene expression among strains, we checked whether the proportion of methylated profiles differently occurred between protein coding and noncoding regions. Accordingly, to MeStudio software (Riccardi et al. 2022), the frequency of methylated motifs was computed along sense and antisense DNA strands, considering four DNA regions: (i) coding (CDS), (ii) noncoding (nCDS), (iii) intergenic (tIG), and (iv) upstream (US) of genes regions (as defined in supplementary fig. S3, Supplementary Material online). Raw data are reported in supplementary dataset S4, Supplementary Material online. When considering the mean methylation frequencies for each DNA region, tIG showed the highest number of DNA methylated motifs with statistically significant differences (one-way ANOVA and Dunn's post hoc test *P* < 0.05, see supplementary dataset S4, Supplementary Material online for more details). tIG was the feature showing the highest variance indicating a clear difference across genomes in the patterns of DNA methylation frequencies compared to the other DNA regions (Fig. 1a).

Moreover, we evaluated the strain-to-strain variation across DNA regions calculating the normalized root-mean-square deviation (NRMSD) (Fig. 1b and c) over the frequencies of methylation; lower values of NRMSD correspond to lower interstrain variability. The NRMSD values depicted in the heatmaps presented in Fig. 1b and c are subject to scaling procedures, specifically row-wise and column-wise scaling. This approach is employed to accentuate distinct patterns of motifs across genomic features, considering the high order of magnitude in-between data. Row-wise scaling enables the emphasis of such patterns. The cell cycle-related DNA methylated motif GANTC exhibited the lowest values, indicating a nearly negligible inter-DNA region variability. The other motifs exhibited a NRMSD of methylated fractions 100 times higher than GANTC, which indicates uneven distribution among DNA regions (CDS, nCDS, tIG, and US) and may suggest that differential DNA methylation of such motifs is involved in methylation-mediated variability of gene expression among strains. The high variability reflected by the NRMSD values results in three distinct clusters of motifs (Fig. 1b) exhibiting notably low values (from GANTC to YCGGCCGRV), significantly high values (from RGATCY to TGGGCA), and values approximating to zero (from ACGGAG to TCGWCGA). Column-wise scaling serves to center DNA regions as variables (Fig. 1c). Notably, the tIG region manifested the highest variability in NRMSD values. A substantial cluster of methylated motifs (12) was characterized by NRMSD values exceeding 1, indicative of a pronounced variability in their frequency among strains. Conversely, the remaining motifs exhibited the lowest values, suggesting a more similar presence across strains.

**Fig. 4.**
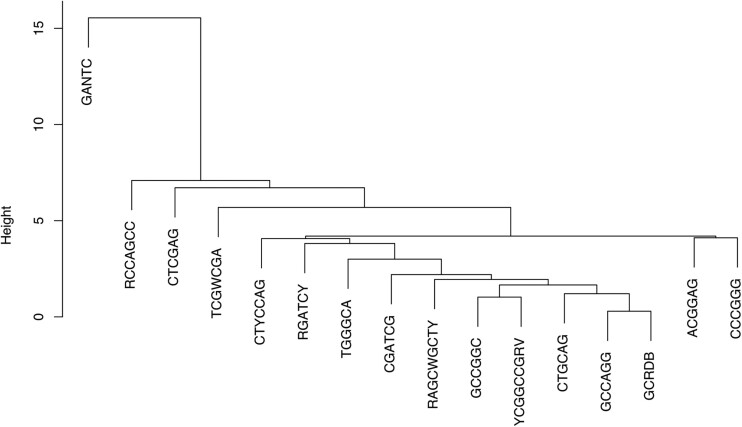
Closely related motifs share similar methylation patterns. Dendrogram representing scaled Euclidean distances (method hclust “complete”) for the tIG methylated motifs. The GANTC motif displays a peculiar methylation pattern when compared to the other motifs, which cluster all together.

To further inspect the uneven distribution of DNA methylated motifs in tIG intergenic regions, we performed a cluster analysis of the methylation frequencies of the motifs (Fig. 4). This analysis aimed to ascertain whether motifs in close proximity, as determined by Euclidean distance (i.e. showing similar methylation frequency patterns), exhibited similarities in terms of the methylated genes with which they are associated. As expected GANTC was a standalone motif, and it was branching out with respect to the other motifs which formed different groups. We found clustering of motifs as ACGGAG with CCCGGG, GCRDB with GCCACG, and GCCGGC with YCGGCCGRV, which suggest they may have similar roles on the regulation of gene expression. These results again indicate that methylation frequency is not evenly distributed across DNA regions, then supporting the hypothesis that the presence of specific DNA methylation patterns could have a gene expression regulatory role. When considering the rightmost genes to the tIG DNA region for the methylated motifs which are clustering together (i.e. GCRDB and GCCAGG), we noticed associated (downstream) genes such as those coding for IS3 and IS5 family transposase (*ISSme1* and *ISRtr4*), nodulation protein D2, or the glycine cleavage system transcriptional activator (supplementary dataset S6, Supplementary Material online). The observation that motifs which cluster together also display methylation in the vicinity of the same orthologous genes could imply that these methylated motifs may share similar functional roles within this shared gene set. For GANTC, the top methylation hits were related to genes which are involved in the cell cycle regulation, such as those encoding the chromosomal replication initiator protein DnaA, single-stranded-DNA-specific exonuclease RecJ, and others, which makes sense considering their role in cell cycle progression.

### Epigenomic Profiles Are Mainly Similar Across Replicons

**Fig. 5.**
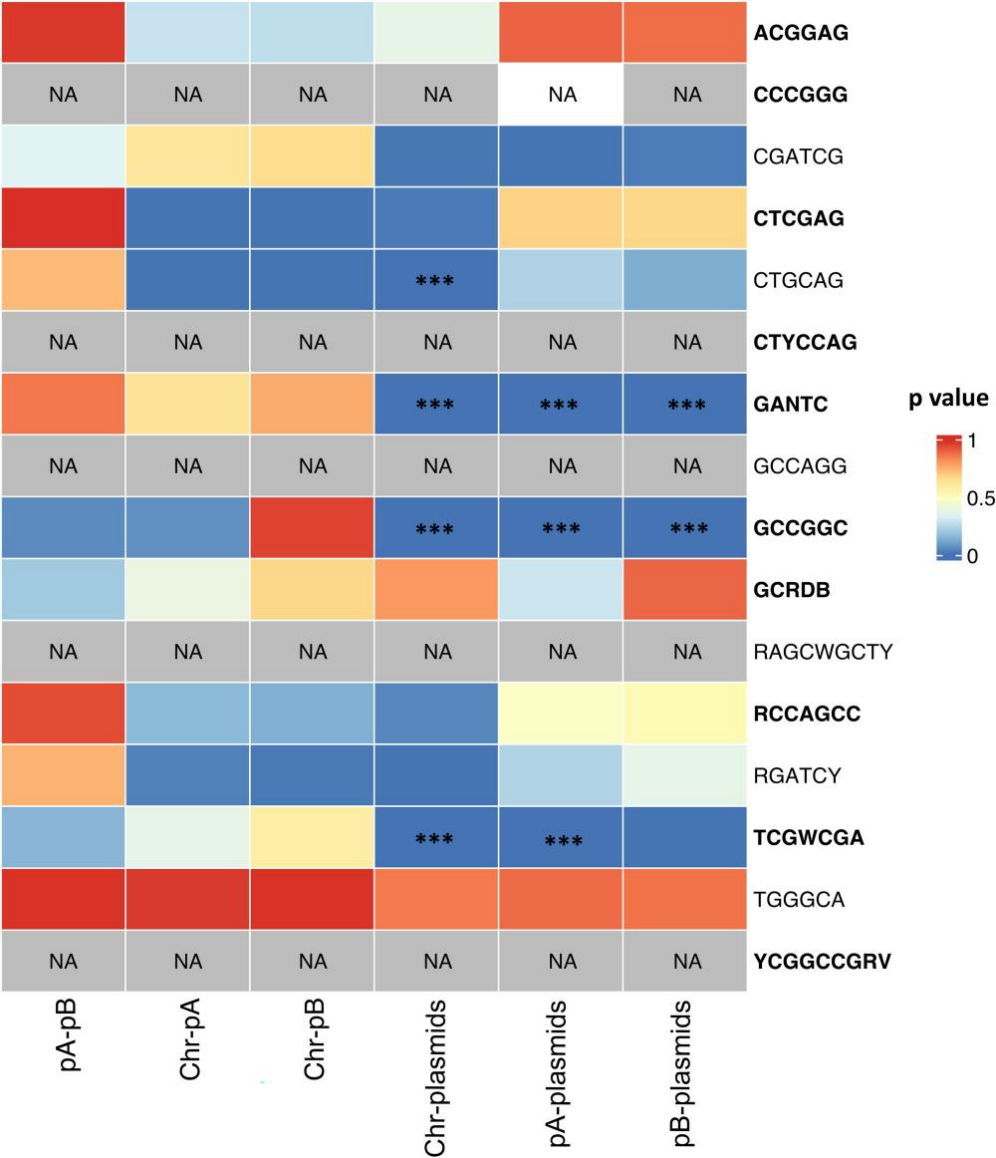
Plasmids are differentially methylated with respect to the rest of the genome. Heatmap of Dunn's multiple comparison test. Asterisks indicate statistically significant differences (*P* < 0.001). pSymA, pSymB, and chromosome are, respectively, indicated in the figure as pA, pB, and Chr.

Since *S. meliloti* harbors a multipartite genome structure, which is connected to a functional specialization of the different replicons (chromosome, pSymB chromid, and pSymA megaplasmid) and to different evolutionary dynamics (Galardini et al. 2013; diCenzo et al. 2014; Schulte et al. 2022) we evaluated if DNA methylation profiles may reflect such intragenomic differences among replicons. The frequency of DNA methylated motifs along the 21 genomes of *S. melilot*i strains was compared among replicons (Fig. 5; supplementary dataset S4, Supplementary Material online). Out of the 16 motifs identified, four showed high statistically significant differences (Dunn's test *P* < 0.001) in the frequency of methylation between plasmids and the rest of the replicons. Interestingly, the methylation frequencies of plasmids displayed differences even for GANTC motifs, suggesting an uncoupling of the control of DNA duplication for such additional replicons. This possible uncoupling of DNA replication from cell cycle control in the divided *S. meliloti* genome was previously suggested for the megaplasmid pSymA in terminally differentiated cells (bacteroids) inside root nodules (diCenzo et al. 2022).

## Conclusion

Genome-wide DNA methylation patterns are key elements in the genotype-to-phenotypes transition, having profound consequences on adaptation and phenotypic plasticity (Fu et al. 2012). In prokaryotes, DNA methylation can have several roles, from DNA repair mechanisms to cell cycle control, self-DNA recognition, and transcriptional regulation (Oliveira and Fang 2021). Mirroring these multiple roles of DNA methylation, the phylogeny of DNA MTasess is also complex, including both vertical and horizontal transmission. In principle, this may give rise to both DNA methylation patterns shared among strains and to strain-specific DNA methylation profiles, resulting from genes harbored in the core and in the shell genome fraction, respectively. In agreement with such hypotheses, evidence from our data on *S. meliloti* indicates that a shared by all (core) and not universally shared (shell) set of DNA methylation profiles exist. Such an epigenomic landscape, with a core and shell epigenome, is in line with the open pangenome of this species (Galardini et al. 2013), consisting of core and shell genome fractions. However, the phylogenetic trees on core and shell genomes of the *S. meliloti* strains only partially mirror epigenome similarities, suggesting that epigenome profiling (presence and frequency of methylated DNA motifs) cannot be simply explained by evolutionary relatedness. Indeed, considering the possibility that a substantial fraction of the methylated motifs may derive from HGT events (being the motifs occurring in a part only of the strains) we should consider the differential influence that acquired DNA methylation profiles may have on the subsequent acquisitions of DNA. When DNA methylation is related to R–M systems, the acquisition by HGT of a novel R–M system, clearly create a barrier to gene flow. Under these conditions, the acquisition of additional novel DNA (and of potentially novel DNA MTases and consequently new DNA methylation profiles) is strongly reduced (Shi et al 2022). This may bring to a reduction of gene flow among strains with different methylation profiles, favoring the maintenance of cohesive population structures, which in turn can give rise to a new lineage in the population (Oliveira and Fang 2021). On the contrary, when novel DNA MTases (and then novel DNA methylation profiles), unlinked to the R–M systems, are acquired, they have potentially no effect over gene flow, hence novel DNA acquisitions. Consequently, we can reason that the pattern of the shell epigenome is the result of the timing of acquisition of DNA MTases unlinked (“neutral” to the HGT) and linked (“negative” to the HGT). This model will lead to a mirroring of the shell genome phylogeny only for those branches where mostly “neutral” profiles have been acquired. The experimental evidence of differences among strains for gene transfer frequencies in our study may suggest the presence of some “negative”-to-HGT methylation profiles. However, from our data, we cannot rule out which profile can be more related to differences in gene transfer among the selected strains. Still concerning the relationships between HGT and DNA methylation, we inspected the multireplicon structure of *S. meliloti* genome with respect to the pattern of methylation of the chromosome, the chromid pSymB, and the symbiotic megaplasmid pSymA, the three resident replicons of *S. meliloti* (Harris and Goldman 2020). Evolutionary analyses on *S. meliloti* genomes clearly indicated that pSymA megaplasmid is an alien element which was recently acquired by the resident genome, while pSymB is a chromid, which stabilizes in the ancestral *S. meliloti* genome (Galardini et al. 2013; diCenzo et al. 2014; Herman and Sultan 2016; Harris and Goldman 2020). The other additional plasmids can be postulated to be the most recently acquired genomic elements. In line with this timing of replicon acquisition are the statistically significant differences between the plasmid replicons and the rest of the genome. This evidence possibly indicates that the DNA methylation status could represent another parameter regulating the genomic integration in multipartite genomes. There are recent indications that HGT elements (i.e. plasmids) are less targeted by R–M systems (Riley et al. 2023). We can hypothesize that the plasmid elements may avoid the action of R–M systems because of their different methylation abundance of specific target motifs. Moreover, DNA regions acquired through HGT (Jeltsch 2003; Harris and Goldman 2020), tend to have low expression levels and slowly integrate into the transcriptional cellular network (Galibert et al. 2001; Lercher and Pál 2008). This low level of expression can be related to the presence of xenogeneic silencers, such as MucR in *Sinorhizobium fredii* (Jiao et al. 2022), which preferentially targets regions with high AT content. We may speculate that the different methylation frequency for some motifs in the plasmid replicons may partially reflect a stronger control of gene expression operated during the growth conditions used herein. A hypothesis on the role of the DNA methylation on gene expression regulation was drawn from the results obtained when measuring the frequency of methylation in the different DNA regions, as well as the strain-to-strain variability. Since large strain-to-strain variation in transcriptomes of *S. meliloti* strains has been previously shown and related to genotypic interaction with the host plant (Fagorzi et al. 2021), we cannot a priori exclude that strain-to-strain epigenomic differences can have an influence over the observed transcriptomic variations. Additional investigations of methylation patterns under different growth conditions coupled with transcriptomic analyses will be performed to sustain the hypothesis of epigenomic deriving phenotypic plasticity and transcriptional variation in *S. meliloti* strains.

In conclusion, here we showed for the first time that a large pan-epigenome exists in bacteria. Moreover, using *S. meliloti* (a nitrogen fixing, multipartite genome model organism) as a study model, we found that replicons share methylation profiles only partially mirroring replicon phylogenies, and we recognized that DNA regions (coding and noncoding parts) display a differential methylation also highly variable among strains. These data allow formulating the hypothesis that DNA methylation may play a role in gene regulation and in phenotypic variation at the strain level. Investigation on DNA MTases mutants and additional analyses of genome-wide methylation in different culture conditions are needed for thoroughly testing this hypothesis.

## Supplementary Material

evae245_Supplementary_Data

## Data Availability

Sequencing data are deposited under NCBI BioProject Accession PRJNA681719. Analyzed data (supplementary datasets S1–S6, Supplementary Material online) are available on the Zenodo repository at the following doi: 10.5281/zenodo.8074352. Custom scripts and MeStudio software can be found at the GitHub repository: https://github.com/combogenomics/MeStudio.
