# Supplementary material for: The Complex Epigenetic Panorama in the Multipartite Genome of the Nitrogen-Fixing Bacterium Sinorhizobium meliloti
Source: Genome Biol Evol. 2025 Jan 9;17(1):evae245. doi: 10.1093/gbe/evae245 (PMC11711589; doi:10.1093/gbe/evae245)
Supplement: evae245_Supplementary_Data [file evae245_supplementary_data.zip › Supplementary information file_final_check.pdf]

The complex epigenetic panorama in the multipartite genome of the nitrogen-fixing bacterium *Sinorhizobium meliloti*

Iacopo Passeri, Lisa Cangioli, Marco Fondi, Alessio Mengoni, Camilla Fagorzi**

Department of Biology, University of Florence, via Madonna del Piano 6, 50019, Sesto Fiorentino, Italy

**Corresponding author: alessio.mengoni@unifi.it; camilla.fagorzi@unifi.it*

Supplementary information

Supplementary Dataset S1. Description of all the analysed strains including all the information (origin, host plant, genomic characteristics) (.xlsx file).

Supplementary Dataset S2. Genome coverage data and assignment of contigs to *S. meliloti* 1021 genome replicons. MS Excel file format (.xlsx file).

Supplementary Dataset S3. Number motifs and methylated motifs identified and their ratio. Data on number of motifs (RAW), number of methylated motifs (METH), fraction of methylated motifs (RATIO) related to functional features (CDSid, tIG and US sequences) and to replicons (CHR, chromosome; PA, pSymA; PB, pSymB and plasmids) are reported. Red color in columns from "plasmids" indicate missing data (i.e., absence of replicons other than chromosome, pSymA, pSymB) (.xlsx file).

Supplementary Dataset S4. Folder containing summary pangenome statistics, core and accessory orthologs, alignments and R script (RAR archive).

Supplementary Dataset S5. CD-HIT cluster analysis. Raw table containing the found 31 clusters of DNA MTases with 0.7 threshold identity (.xls file).

Supplementary Dataset S6. List of the top-5 rightmost genes to the tIG DNA region for the methylated motifs which are clustering together in the dendrogram of Figure 4 (i.e., GCRDB and GCCAGG) (.xlsx file). N/A indicates that the motif is not methylated.

Putative replication initiation protein RepC coding genes

Table S1. Locus tag (GenBank codes) of genes coding for RepC protein identified by genome annotation performed by the NCBI Prokaryotic Genome Annotation Pipeline (PGAP).

**Strain/GenBank locus tags (repC gene)**						
1A42	I7G55_31090	I7G55_31830				
4B41	I7I49_19270	I7I49_24670				
7B22	I7G59_00015	I7G59_20250	I7G59_32180			
8A52	I7F98_01675	I7F98_26050				
AE608H	I7G86_26910	I7G86_27090	I7G86_35295			
AK58	LOF18_00350	LOF18_00570	LOF18_12670	LOF18_14870		
AK75	LOF12_24775	LOF12_26095	LOF12_32700			
AK83	LOF17_00050	LOF17_00235	LOF17_14590	LOF17_14705	LOF17_15940	
AL703GG	I7G01_00005	I7G01_07565	I7G01_13345	I7G01_15370	I7G01_15915	I7G01_23550
AL703H	I7G85_20290	I7G85_24345	I7G85_25215	I7G85_25655		
AO641M	I7F95_03770	I7F95_10950	I7F95_11365	I7F95_1954		
AO643DD	LOF23_19910	LOF23_27580	LOF23_35445			
BL225C	LOF16_05515	LOF16_15565				
CE480L	I7G00_19045	I7G00_20425	I7G00_34790			
CL374FF	I7F97_00175	I7F97_15100	I7F97_15665	I7F97_31915		
CO431A	LOF22_25585	LOF22_33340				
CO438LL	LOF28_10065	LOF28_10280	LOF28_17945	LOF28_18695		
H1	I7G60_16970	I7G60_17440	I7G60_26575	I7G60_27510		
NRG-185	I7F13_00035	I7F13_07680	I7F13_15140	I7F13_16300		
SM11	LOF24_19230	LOF24_26785	LOF24_28185	LOF24_28350		
T073	I7F30_18610	I7F30_19020	I7F30_32420			

Methylated motifs identified

The analysis of SMRT sequencing genome data with SMRT Link software ver. 8.0.0.80529 using default options identified 27 methylated motifs (Table S2A). Some of the motifs (shared colour) were further collapsed together since they share a common core motif (Table 2B).

Table S2. Raw (A) and collapsed (B) methylated motifs. Same colours indicated raw motifs collapsed into a single core motif.

A)

Raw motifs
ACGGAG
BNNCGATCGV
BYCGATCG
CCCGGG
CGATCGV
CTCGAG
CTYCCAG
DCTGCAGGS
GANTC
GCCAGG
GCCGGCH
GCCGGCYD
GCRDB
GNCGATCGVC
RAGCWGCTY
RCCAGCC
RCGATCGGC
RCTGCAGGS
RGATCY
SCTCGAG
TCGWCGA
TGGGCA
VGCCGGCCC
VNCGATCGV
YCGATCGD
YCGGCCGRV
YCTGCAG

B)

Motif	Type

ACGGAG	m6A
CGATCG	m6A
CCCGGG	m4C
CTCGAG	m6A
CTYCCAG	m6A
CTGCAG	m6A
GANTC	m6A
GCCAGG	m6A
GCCGGC	m4C
GCRDB	m4C
RAGCWGCTY	m4C
RCCAGCC	m4C
RGATCY	m6A
TCGWCGA	m4C
TGGGCA	m6A
YCGGCCGRV	m4C

Putative DNA methyltransferases

Table S3. Locus tag (GenBank codes) of DNA methyltransferases identified by genome annotation performed by the NCBI Prokaryotic Genome Annotation Pipeline (PGAP).

**Strain/GenBank locus tags**									
1A42	I7G55_00850	I7G55_04930	I7G55_10975	I7G55_10985	I7G55_30135				
4B41	I7I49_01000	I7I49_06525	I7I49_06535	I7I49_12810	I7I49_16825	I7I49_25660	I7I49_29760		
7B22	I7G59_01700	I7G59_05600	I7G59_06395	I7G59_06405	I7G59_09770	I7G59_19945	I7G59_25705		
8A52	I7F98_02635	I7F98_19595	I7F98_23450	I7F98_25655					
AE608H	I7G86_05530	I7G86_05560	I7G86_07470	I7G86_07480	I7G86_13825	I7G86_18415	I7G86_29425	I7G86_33635	
AK58	LOF18_01550	LOF18_05945	LOF18_13010	LOF18_13015	LOF18_16435	LOF18_20780	LOF18_26225	LOF18_33095	
AK75	LOF12_00870	LOF12_02240	LOF12_04640	LOF12_22340	LOF12_26060				
AK83	LOF17_06465	LOF17_07730	LOF17_21370	LOF17_21380	LOF17_29840	LOF17_32675	LOF17_34045		
AL703GG	I7G01_05265	I7G01_08645	I7G01_15395	I7G01_27770	I7G01_29440	I7G01_32085			
AL703H	I7G85_00865	I7G85_04600	I7G85_11135	I7G85_32280					
AO641M	I7F95_12345	I7F95_16635	I7F95_19520	I7F95_20565	I7F95_22230	I7F95_24885	I7F95_31755		
AO643DD	LOF23_01005	LOF23_01035	LOF23_07360	LOF23_08780	LOF23_11500	LOF23_12275	LOF23_18640	LOF23_29880	LOF23_34140
BL225C	LOF16_06465	LOF16_23090	LOF16_24725	LOF16_24735	LOF16_28720	LOF16_31075	LOF16_32450		
CE480L	I7G00_06770	I7G00_13630	I7G00_17750	I7G00_19200	I7G00_21390				
CL374FF	I7F97_08515	I7F97_12800	I7F97_15640	I7F97_16780	I7F97_23625	I7F97_26275	I7F97_27930		
CO431A	LOF22_00865	LOF22_02240	LOF22_04595	LOF22_08580	LOF22_08590	LOF22_10220	LOF22_26565		
CO438LL	LOF28_11245	LOF28_15495	LOF28_20145	LOF28_26550	LOF28_29285	LOF28_30775			
H1	I7G60_07150	I7G60_12280	I7G60_16015	I7G60_24220					
NRG185	I7F13_06620	I7F13_20825	I7F13_21840	I7F13_23250	I7F13_23285	I7F13_28155	I7F13_31975		
SM11	LOF24_12700	LOF24_12730	LOF24_14415	LOF24_16980	LOF24_18355	LOF24_21550	LOF24_25795	LOF24_28160	
T073	I7F30_07070	I7F30_07080	I7F30_07745	I7F30_11480	I7F30_15215	I7F30_31470			

Figure S1. **Clustering of strains based on the sharing of DNA MTases orthologous groups.**

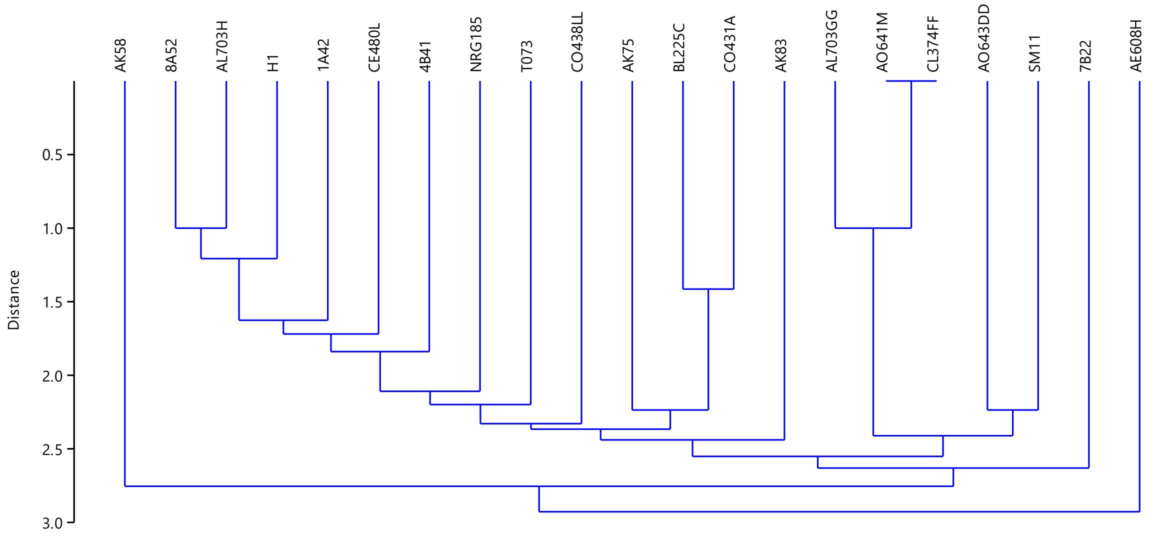

Plasmid transfer experiment

The artificial plasmid pMP7604 was used as probe for gene transfer experiments. Plasmid pMP7604 is a broad host range plasmid containing constitutively expressed mCherry gene and tetracycline resistance (Lagendijk et al., 2010). pMP7604 sequence is based on pME6031 vector (AddGene code V004776#) where the monomeric red fluorescent protein gene (GenBank accession number AY678264.1) downstream to a *tac* promoter (5'-TTGACAATTAATCATCGGCTCGTATAATG-3') have been cloned. The plasmid was inserted into *S. meliloti* strains by biparental mating using the rifampicin-resistant derivatives of *S. meliloti* strains as recipient strains, and *E. coli* S17-1 harboring pMP7604 as donor. The plasmid pMP7604 was then extracted from TY grown cultures of each *S. meliloti* strain with Qiagen miniprep kit. Quantity and quality of the plasmid DNA was estimated by 1% agarose gel electrophoresis in TAE buffer (40 mM Tris, 20 mM acetic acid, 1 mM EDTA). Then plasmid was extracted from each derivative *S. meliloti* strain culture and used in a pairwise combination to electroporate *S. meliloti* cells from the same and from the other 4 strains. DNA acquisition ratios were then computed as ratio between the efficiency of transformation (CFU/µg DNA) with not-self and with self-DNA. Preparation of electrocompetent *S. meliloti* cells vector was carried out as described in (Ferri et al., 2010). Briefly cells were grown in 100 ml TY medium at 30°C with shaking (130 rpm) until late exponential phase was reached. Cultures were cooled on ice for 15 minutes and centrifuged at 8000 rpm at 4°C for 10 minutes. Pellets were washed three times in ice cold MilliQ water and, finally, resuspended into 500 µl 10% glycerol to obtain 50 µl portions containing $1x10^8$ cells each. Electroporation was performed with $1x10^8$ *S. meliloti* competent cells and 1 µl vector (10-80 ng), according to the following settings: Voltage = 2.10 kV, Capacitance = 25 mF, Resistance = 200 Ohm.

In Table S4 the motifs identified in the *S. meliloti* genomes analyzed and present in pMP7604 are reported. The efficiency of transformation (CFU/µg DNA) with not-self and with self-DNA is reported in Table S5. Figure S1 reports the normalized efficiency of transformation against self-DNA (log10 scale) and Table S6 the p-values of pairwise contrasts.

Figure S2. **Pairwise transformation normalized efficiency.** Matrix of log10 fold change of DNA acquisition ratios against self-DNA (rows donors, columns recipient strains). Donors are indicated with the prefix “pMP7604_Me”.

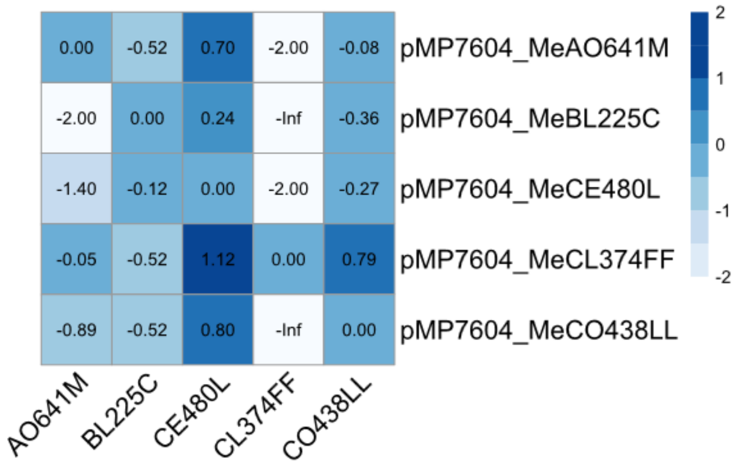

Table S4. Occurrences of motifs in the pMP7604 sequence.

Motif	Occurrence in pMP7604
ACGGAG	2
CGATCG	3
CCCGGG	2
CTCGAG	1
CTYCCAG	2
CTGCAG	2
GANTC	24
GCCAGG	7
GCCGGC	28
GCRDB	258
RAGCWGCTY	0
RCCAGCC	3
RGATCY	7
TCGWCGA	1
TGGGCA	2
YCGGCCGRV	4

Table S5. **Gene transfer frequencies**. The table reports the details of the electroporation experiments performed. Number of recovered transformants and normalized efficiency of transformation (against self-DNA) are reported. Data are average of three replicate experiments. “p” indicates the donor (plasmid). The first two letters of strain codes are indicated.

**Donor**	**Recipient**	**Mean number of colonies colonies**	**Mean efficiency**
pAO	AO	15.0	7.50E-09
pBL	AO	0.7	1.11E-10
pCE	AO	2.7	3.33E-10
pCL	AO	6.7	6.67E-09
pCO	AO	1.0	1.00E-09
pAO	BL	0.7	3.33E-10
pBL	BL	6.7	1.11E-09
pCE	BL	2.0	2.50E-10
pCL	BL	0.3	3.33E-10
pCO	BL	0.3	3.33E-10
pAO	CE	10.0	5.00E-09
pBL	CE	10.3	1.72E-09
pCE	CE	8.0	1.00E-09
pCL	CE	13.3	1.33E-08
pCO	CE	6.3	6.33E-09
pAO	CL	1.0	5.00E-10
pBL	CL	1.0	1.67E-10
pCE	CL	2.0	2.50E-10
pCL	CL	40.7	4.07E-08
pCO	CL	0.0	0.00E+00
pAO	CO	3.3	1.67E-09
pBL	CO	5.3	8.89E-10
pCE	CO	8.7	1.08E-09
pCL	CO	12.3	1.23E-08
pCO	CO	2.0	2.00E-09

Table S6. **Statistical significance of pairwise comparisons of transformation efficiencies**. Symmetric diagonal matrix of p-values from Dunn's post hoc test. Highly significant contrasts (p<0.01) are in bold. "p" indicates the donor (plasmid). The first two letters of strain codes are indicated.

	pAO-AO	pBL-AO	pCE-AO	pCL-AO	pCO-AO	pAO-BL	pBL-BL	pCE-BL	pCL-BL	pCO-BL	pAO-CE	pBL-CE	pCE-CE	pCL-CE	pCO-CE	pAO-CL	pBL-CL	pCE-CL	pCL-CL	pCO-CL	pAO-CO	pBL-CO	pCE-CO	pCL-CO	pCO-CO
pAO-AO		**0.004**	0.019	0.918	0.077	**0.009**	0.185	0.013	**0.009**	**0.009**	0.778	0.452	0.150	0.763	0.910	0.036	**0.007**	0.013	0.541	**0.002**	0.332	0.112	0.218	0.778	0.259
pBL-AO	**0.004**		0.579	**0.005**	0.259	0.771	0.116	0.686	0.771	0.771	**0.009**	0.032	0.145	**0.001**	**0.005**	0.424	0.836	0.686	**0.000**	0.785	0.054	0.191	0.096	**0.001**	0.077
pCE-AO	0.019	0.579		0.025	0.566	0.792	0.309	0.880	0.792	0.792	0.039	0.112	0.366	**0.008**	0.026	0.807	0.728	0.880	**0.003**	0.408	0.169	0.452	0.267	**0.009**	0.225
pCL-AO	0.918	**0.005**	0.025		0.096	0.012	0.221	0.017	0.012	0.012	0.858	0.516	0.181	0.686	0.993	0.046	**0.010**	0.017	0.475	**0.002**	0.387	0.137	0.259	0.700	0.305
pCO-AO	0.077	0.259	0.566	0.096		0.402	0.658	0.469	0.402	0.402	0.137	0.309	0.742	0.038	0.098	0.742	0.356	0.469	0.017	0.161	0.424	0.858	0.592	0.040	0.522
pAO-BL	**0.009**	0.771	0.792	0.012	0.402		0.201	0.910	1.000	1.000	0.020	0.064	0.243	**0.004**	0.013	0.611	0.933	0.910	**0.001**	0.572	0.102	0.309	0.169	**0.004**	0.140
pBL-BL	0.185	0.116	0.309	0.221	0.658	0.201		0.243	0.201	0.201	0.296	0.566	0.910	0.104	0.225	0.440	0.172	0.243	0.053	0.065	0.721	0.792	0.925	0.108	0.843
pCE-BL	0.013	0.686	0.880	0.017	0.469	0.910	0.243		0.910	0.910	0.027	0.082	0.292	**0.005**	0.017	0.693	0.843	1.000	**0.002**	0.498	0.127	0.366	0.207	**0.005**	0.172
pCL-BL	**0.009**	0.771	0.792	0.012	0.402	1.000	0.201	0.910		1.000	0.020	0.064	0.243	**0.004**	0.013	0.611	0.933	0.910	**0.001**	0.572	0.102	0.309	0.169	**0.004**	0.140
pCO-BL	**0.009**	0.771	0.792	0.012	0.402	1.000	0.201	0.910	1.000		0.020	0.064	0.243	**0.004**	0.013	0.611	0.933	0.910	**0.001**	0.572	0.102	0.309	0.169	**0.004**	0.140
pAO-CE	0.778	**0.009**	0.039	0.858	0.137	0.020	0.296	0.027	0.020	0.020		0.638	0.247	0.560	0.866	0.069	0.016	0.027	0.371	**0.004**	0.492	0.191	0.342	0.572	0.397
pBL-CE	0.452	0.032	0.112	0.516	0.309	0.064	0.566	0.082	0.064	0.064	0.638		0.492	0.292	0.522	0.178	0.053	0.082	0.172	0.016	0.829	0.402	0.631	0.301	0.707
pCE-CE	0.150	0.145	0.366	0.181	0.742	0.243	0.910	0.292	0.243	0.243	0.247	0.492		0.082	0.185	0.510	0.211	0.292	0.040	0.083	0.638	0.880	0.836	0.085	0.756
pCL-CE	0.763	**0.001**	**0.008**	0.686	0.038	**0.004**	0.104	**0.005**	**0.004**	**0.004**	0.560	0.292	0.082		0.679	0.016	**0.003**	**0.005**	0.756	**0.001**	0.204	0.059	0.125	0.985	0.153
pCO-CE	0.910	**0.005**	0.026	0.993	0.098	0.013	0.225	0.017	0.013	0.013	0.866	0.522	0.185	0.679		0.047	**0.010**	0.017	0.469	**0.002**	0.392	0.140	0.263	0.693	0.309
pAO-CL	0.036	0.424	0.807	0.046	0.742	0.611	0.440	0.693	0.611	0.611	0.069	0.178	0.510	0.016	0.047		0.553	0.693	**0.007**	0.283	0.259	0.611	0.387	0.017	0.332
pBL-CL	**0.007**	0.836	0.728	**0.010**	0.356	0.933	0.172	0.843	0.933	0.933	0.016	0.053	0.211	**0.003**	**0.010**	0.553		0.843	**0.001**	0.631	0.085	0.271	0.145	**0.003**	0.118
pCE-CL	0.013	0.686	0.880	0.017	0.469	0.910	0.243	1.000	0.910	0.910	0.027	0.082	0.292	**0.005**	0.017	0.693	0.843		**0.002**	0.498	0.127	0.366	0.207	**0.005**	0.172
pCL-CL	0.541	**0.000**	**0.003**	0.475	0.017	**0.001**	0.053	**0.002**	**0.001**	**0.001**	0.371	0.172	0.040	0.756	0.469	**0.007**	**0.001**	**0.002**		**0.000**	0.114	0.028	0.065	0.742	0.082
pCO-CL	**0.002**	0.785	0.408	**0.002**	0.161	0.572	0.065	0.498	0.572	0.572	**0.004**	0.016	0.083	**0.001**	**0.002**	0.283	0.631	0.498	**0.000**		0.028	0.114	0.053	**0.001**	0.041
pAO-CO	0.332	0.054	0.169	0.387	0.424	0.102	0.721	0.127	0.102	0.102	0.492	0.829	0.638	0.204	0.392	0.259	0.085	0.127	0.114	0.028		0.535	0.792	0.211	0.873
pBL-CO	0.112	0.191	0.452	0.137	0.858	0.309	0.792	0.366	0.309	0.309	0.191	0.402	0.880	0.059	0.140	0.611	0.271	0.366	0.028	0.114	0.535		0.721	0.061	0.645
pCE-CO	0.218	0.096	0.267	0.259	0.592	0.169	0.925	0.207	0.169	0.169	0.342	0.631	0.836	0.125	0.263	0.387	0.145	0.207	0.065	0.053	0.792	0.721		0.130	0.918
pCL-CO	0.778	**0.001**	**0.009**	0.700	0.040	**0.004**	0.108	**0.005**	**0.004**	**0.004**	0.572	0.301	0.085	0.985	0.693	0.017	**0.003**	**0.005**	0.742	**0.001**	0.211	0.061	0.130		0.158
pCO-CO	0.259	0.077	0.225	0.305	0.522	0.140	0.843	0.172	0.140	0.140	0.397	0.707	0.756	0.153	0.309	0.332	0.118	0.172	0.082	0.041	0.873	0.645	0.918	0.158	

Differences in methylation densities across coding and noncoding regions

MeStudio procedure starts with matching the nucleotide motifs to the genomic sequence and map them to the corresponding category, which are extracted from the annotation file (GFF file). Categories are defined as: i) protein-coding genes with accordant (sense) strand (CDS), ii) regions that fall between annotated genes (true intergenic, tIG), iii) regions upstream to the reading frame of a gene, with accordant strand (US) (Figure S2). The CDS feature is defined by the ORF. The tIG is defined as the region between two different ORFs on both strands, as reported in Figure S3. The US region is defined, by default, as the portion of the genome between the end of an ORF and the beginning of the next one (Riccardi et al., 2023).

Results of statistical analysis of the overall densities of methylated motifs for CDS, tIG and US sequences in the *S. meliloti* replicons is reported in Table S7.

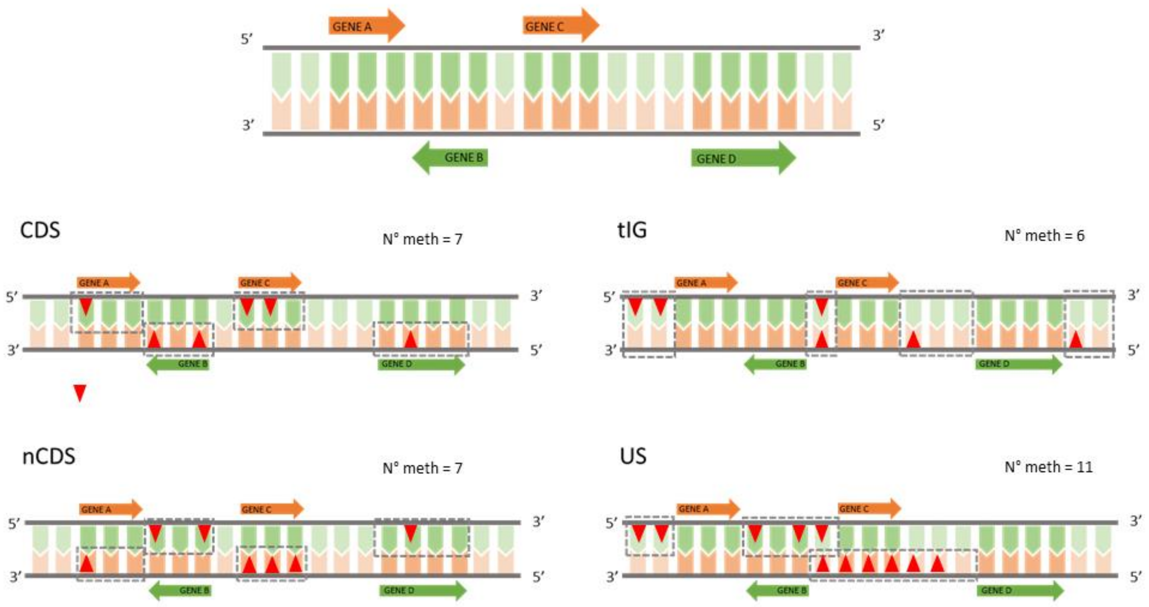

Figure S3. Graphical representation of the used terminology as in MeStudio software. CDS, coding sequence; tIG, intergenic sequence between two genes in opposite directions; US, upstream sequence to a coding sequence, (intergenic sequence between two genes having the same orientation).

References

Ferri, L., Gori, A., Biondi, E. G., Mengoni, A., & Bazzicalupo, M. (2010). Plasmid electroporation of Sinorhizobium strains: The role of the restriction gene hsdR in type strain Rm1021. *Plasmid*. https://doi.org/10.1016/j.plasmid.2010.01.001

Lagendijk, E. L., Validov, S., Lamers, G. E. M., De Weert, S., & Bloemberg, G. V. (2010). Genetic tools for tagging Gram-negative bacteria with mCherry for visualization in vitro and in natural habitats, biofilm and pathogenicity studies. *FEMS Microbiology Letters*. https://doi.org/10.1111/j.1574-6968.2010.01916.x

Riccardi, C., Passeri, I., Cangioli, L., Fagorzi, C., Fondi, M., & Mengoni, A. (2023). Crossing Bacterial Genomic Features and Methylation Patterns with MeStudio: An Epigenomic Analysis Tool. *International Journal of Molecular Sciences*, *24*(1). https://doi.org/10.3390/ijms24010159

Riccardi, C., Passeri, I., Cangioli, L., Fagorzi, C., Mengoni, A., & Fondi, M. (2022). *MeStudio : crossing methylation and genomic features for comparative epigenomic analyses*. 1–10.

Stiens, M., Schneiker, S., Keller, M., Kuhn, S., Pühler, A., & Schlüter, A. (2006). Sequence analysis of the 144-kilobase accessory plasmid pSmeSM11a, isolated from a dominant Sinorhizobium meliloti strain identified during a long-term field release experiment. *Applied and Environmental Microbiology*, *72*(5), 3662–3672. https://doi.org/10.1128/AEM.72.5.3662-3672.2006
